# Abnormal large-scale resting-state functional networks in anti-N-methyl-D-aspartate receptor encephalitis

**DOI:** 10.3389/fnins.2024.1455131

**Published:** 2024-08-19

**Authors:** Xiarong Gong, Libo Wang, Yuanyuan Guo, Yingzi Ma, Wei Li, Juanjuan Zhang, Meiling Chen, Jiaojian Wang, Qiang Meng, Kexuan Chen, Yanghua Tian

**Affiliations:** ^1^Faculty of Life Science and Technology, Kunming University of Science and Technology, Kunming, China; ^2^Department of MR, The First People’s Hospital of Yunnan Province, Kunming, China; ^3^The Second People’s Hospital of Yuxi, The Affiliated Hospital of Kunming University of Science and Technology, Kunming, China; ^4^Department of Neurology, The First Affiliated Hospital of Anhui Medical University, Hefei, China; ^5^State Key Laboratory of Primate Biomedical Research, Institute of Primate Translational Medicine, Kunming University of Science and Technology, Kunming, China; ^6^Yunnan Key Laboratory of Primate Biomedical Research, Kunming, China; ^7^Department of Clinical Psychology, The First People’s Hospital of Yunnan Province, The Affiliated Hospital of Kunming University of Science and Technology, Kunming, China; ^8^Department of Neurology, The First People’s Hospital of Yunnan Province, Kunming, China; ^9^Medical School, Kunming University of Science and Technology, Kunming, China; ^10^Department of Neurology, The Second Affiliated Hospital of Anhui Medical University, Hefei, China

**Keywords:** anti-N-methyl-D-aspartate receptor encephalitis, resting-state fMRI, ICA, GCA, functional connectivity, effective connectivity

## Abstract

**Background:**

Patients with anti-N-methyl-D-aspartate receptor (anti-NMDAR) encephalitis often experience severe symptoms. Resting-state functional MRI (rs-fMRI) has revealed widespread impairment of functional networks in patients. However, the changes in information flow remain unclear. This study aims to investigate the intrinsic functional connectivity (FC) both within and between resting-state networks (RSNs), as well as the alterations in effective connectivity (EC) between these networks.

**Methods:**

Resting-state functional MRI (rs-fMRI) data were collected from 25 patients with anti-NMDAR encephalitis and 30 healthy controls (HCs) matched for age, sex, and educational level. Changes in the intrinsic functional connectivity (FC) within and between RSNs were analyzed using independent component analysis (ICA). The functional interaction between RSNs was identified by granger causality analysis (GCA).

**Results:**

Compared to HCs, patients with anti-NMDAR encephalitis exhibited lower performance on the Wisconsin Card Sorting Test (WCST), both in terms of correct numbers and correct categories. Additionally, these patients demonstrated decreased scores on the Montreal Cognitive Assessment (MoCA). Neuroimaging studies revealed abnormal intra-FC within the default mode network (DMN), increased intra-FC within the visual network (VN) and dorsal attention network (DAN), as well as increased inter-FC between VN and the frontoparietal network (FPN). Furthermore, aberrant effective connectivity (EC) was observed among the DMN, DAN, FPN, VN, and somatomotor network (SMN).

**Conclusion:**

Patients with anti-NMDAR encephalitis displayed noticeable deficits in both memory and executive function. Notably, these patients exhibited widespread impairments in intra-FC, inter-FC, and EC. These results may help to explain the pathophysiological mechanism of anti-NMDAR encephalitis.

## Introduction

Anti-N-methyl-D-aspartate receptor (NMDAR) encephalitis is a type of autoimmune encephalitis. It is characterized by acute changes in behavior, psychiatric symptoms, seizures, and memory problems (Wandinger et al., 2011). It affects mainly adolescent girls and teenagers, with a significant impact on their physical and mental health. The etiology of the disease has been clearly identified as being caused by anti-NMDAR antibody-induced downregulation of NMDAR neuronal function. NMDAR hypofunction may induce the elimination of synaptic activity mediated by NMDAR, resulting in impairments in memory, cognition, and behavior. NMDAR, as a subtype of glutamate receptor, widely exists in multiple brain regions. The great heterogeneity of the clinical manifestations of anti-NMDAR encephalitis, associated with the widely distribution of NMDAR, may leads to difficulties in its early diagnosis. The incidence of anti-NMDAR encephalitis has been on the rise for many years, and it is the most common type of autoimmune encephalitis, so it is of great significance to study its neurobiological mechanism for its early detection and treatment.

Although patients with anti-NMDAR encephalitis experience severe clinical symptoms, 50–80% of patients show normal on routine MRI (Heine et al., 2015; Graus et al., 2016), leading to a clinical radiology paradox. We hypothesize that patients with anti-NMDAR encephalitis may exhibit underlying functional alterations imperceptible to the naked eye. Resting-state functional MRI (rs-fMRI) can obtain functional connectivities (FCs) by estimating paired associations of blood oxygen level dependent (BOLD) activity between brain regions (Chen et al., 2024; Yu et al., 2024). Compared to task-state fMRI, rs-fMRI is easier to obtain. Previous fMRI studies have shown that abnormal inter-FCs is associated with disease severity, duration and cognitive symptoms in patients with anti-NMDAR encephalitis (Finke et al., 2013; Peer et al., 2017; Gibson et al., 2019, 2020; Heine et al., 2021; von Schwanenflug et al., 2022). However, it is still unknown that whether intra-FC within the RSNs is affected and how large-scale brain network connectivity is disrupted in anti-NMDAR encephalitis.

Spatial Independent component analysis (ICA), as one of the common methods for fMRI analysis, is a data-driven method based on blind source separation algorithm (Bell and Sejnowski, 1995). ICA decomposes multiple fMRI data to obtain distinct FC patterns in each subject. Next, specific spatial maps and associate time courses for all subjects were extracted and compiled into a single four-dimensional file, containing individual component maps for each subject. Subsequently, the four-dimensional file was split into the three-dimensional file. Finally, differences in FC within and between RSNs were obtained by nonparametric analysis. ICA requires no prior knowledge, however, it ignores the directionality of FC between RSNs (Smitha et al., 2017).

Effective connectivity (EC) may offer additional information about the directionality of information flows within RSNs, using data on time-lag relationships between RSNs (Stephan and Friston, 2010). GCA is one of the methods used to study EC. It is used to analyze rs-fMRI data in a variety of healthy and disease brain networks (Wen et al., 2013; Zhang et al., 2015, 2017). To our knowledge, it is unclear whether directional changes of information flow in anti-NMDAR encephalitis. This approach combined ICA and GCA helps to understand the FCs and the directionality of information flows between brain regions.

In this study, we combine ICA and GCA to analyze rs-fMRI data from 25 patients with anti-NMDA receptor encephalitis and 30 HCs. We compared the FC differences within and between networks among the two groups. Additionally, we examined the EC differences using GCA between the patients and HCs.

## Materials and methods

### Subjects

The study was approved by the ethics committees of Anhui Medical University. Written informed consent was obtained from all participants. All participants both patients with anti-NMDAR encephalitis and HCs were recruited from the First Affiliated Hospital of Anhui Medical University in Hefei, China. It included a total of 25 patients with anti-NMDAR encephalitis and 30 HCs (see Table 1; Supplementary Table S1). Seizures were observed in 17 patients, and psychiatric symptoms were observed in 8 patients. In addition, some patients showed somnipathy (24%), phonism (0.08%) and so on. Nearly all patients received first-line immunotherapy, including corticosteroids, intravenous immunoglobulin, or a combination of these treatments (Supplementary Table S1). The inclusion criteria for patients were as follows: (1) Patients all met the diagnostic criteria for anti-NMDAR encephalitis (Graus et al., 2016). (2) Patients all exhibited typical clinical features and tested positive for NMDAR antibodies in their cerebrospinal fluid (CSF). (3) All participants all underwent a routine MRI and rs-fMRI. (4) They all underwent neuropsychiatric tests. Exclusion criteria for both patients and HCs were as follows: (1) poor-quality patient images; (2) a history of major brain trauma and surgery, craniocerebral disease, arteriovenous malformation, or other neuropsychiatric diseases that may affect intelligence and (3) contraindications to MRI examination.

**Table 1:** Demographic, clinical date of the anti-NMDAR encephalitis patients and healthy controls (HCs).

Subjects	Patients with anti-NMDAR encephalitis	HCs	*p*-value
Number of subjects	25	30	
Gender (male: female)	12/13	13/17	0.832
Age (mean ± SD)	30.20 ± 12.13	35.23 ± 15.08	0.176
Years of education (mean ± SD)	10.90 ± 3.83	9.88 ± 4.32	0.358
PSQI	1 (0, 2)	1 (0, 4)	0.636
HDRS	1 (0, 3)	0 (0, 2)	0.4652
HARS	2 (1, 5)	1 (0, 4)	0.0924
MoCA scores	24 (21, 27)	29 (26, 29)	<0.001
**WSCT score**			
Correct numbers	88.5 (66, 100)	99 (95, 101)	0.018
Perseverative errors	21.5 (12, 35)	11 (10, 15)	0.006
Correct categories	4 (3, 9)	9 (7, 9)	0.002
Random errors	39.5 (21, 62)	19 (14, 29)	0.003

The normality distribution test was performed on all data. The *p*-values were obtained by a Chi-square test for gender, and two-sample *t*-tests for age and education level, Mann–Whitney tests for MoCA scores, HAMA scores, HAMD scores, and WCST score. HDRS, Hamilton Depression Rating Scale; HARS, Hamilton Anxiety Rating Scale; WCST, Wisconsin Card Sorting Test; PSQI, Pittsburgh sleep quality index.

All HCs presented normal head MRI, without any history of neurological or psychiatric disorders. The severity of depressive and anxiety symptoms was measured using the Hamilton Depression Rating Scale (HDRS) and the Hamilton Anxiety Rating Scale (HARS), respectively. Cognitive assessment was performed on all participants using the Montreal Cognitive Assessment (MoCA) scores (Supplementary Table S1). The Wisconsin Card Sorting Test (WCST) was used to assess executive functions in patients with anti-NMDAR encephalitis (Supplementary Table S2). Continuous variables were expressed as mean ± standard deviation or the median with an interquartile range (25, 75%) based by a normal distribution. The two-sample *t*-tests or Mann–Whitney tests were utilized for analyzing continuous variables, while the chi-square test was used for gender.

### MRI data acquisition

MRI data were collected from a 3T GE MR scanner (Discovery 750; GE Healthcare, Milwaukee, WI). rs-fMRI images were acquired using echo-planar imaging with the following parameters: repetition time (TR) = 2,400 ms, field of view (FOV) = 192 × 192 mm^2^, echo time (TE) = 30 ms, 46 slices, slice thickness = 3 mm, voxel size = 3 × 3 × 3 mm, flip angle = 90°, matrix size: 64 × 64, acquisition time = 521 s.

### rs-fMRI data processing

The rs-fMRI data were preprocessed using DPARSFA (Data Processing Assistant for Resting-State fMRI Advanced Edition).^1^ The rs-fMRI data underwent preprocessing, which involved removing the first 10 volumes, performing slice timing correction, head motion correction, spatial normalization, removal of linear drift, and smoothing with a 6 mm Gaussian kernel in a sequential manner. Furthermore, the time series of independent components (ICs) were further detrended, despiked, and filtered with a band-pass of 0.01–0.1 Hz.

### Processing of spatial group ICA

To identify distinct resting-state components in all participants, spatial group ICA was conducted using the GIFT toolbox (Erhardt et al., 2011; Calhoun and Adali, 2012). The rs-fMRI data underwent dimensionality reduction, independent component (IC) identification, and inverse reconstruction. To obtain meaningful ICs, the following criteria were applied: each RSN had to be located in the gray matter and have minimal overlap with white matter structures, ventricles, and blood vessels. Ultimately, 24 functionally related ICs were obtained. Each IC mask was obtained using one-sample *t*-tests, with a threshold of *z* > 1.96 to identify regions of peak activation clusters. The cluster size was set at 30. The ratio weight of each RSN in each function network was determined, and the network with the largest weight was the network corresponding to that IC (see Supplementary Table S3).

To determine group differences between patients and HCs, two-sample *t*-tests were performed for each IC. A threshold corrected for AlphaSim estimate at *p* < 0.05 was used to identify significant changes in FC within each IC, with a cluster forming threshold at voxel-level *p* < 0.001.

### Analyses of inter-FCs between RSNs

For each subject, Pearson correlation coefficients were calculated between each pair of the 24 ICs. To enhance normal distribution, the correlation coefficients were transformed into *z* values using Fisher’s *r*-to-*z* transformation. A 24 × 24 matrix for each individual was obtained. To find the significant alterations, a two-sample *t*-test was conducted to compare the inter-FC length between the two groups. A significance threshold of *p* < 0.05 was set, and multiple comparison correction was applied using Bafferoni corrects.

### Analyses of ECs between RSNs

The causal interaction between each pair of the 24 ICs were identified using GCA. GCA was applied using the Causal Connectivity Toolbox (Seth, 2010). It may reflect the strength and direction of FCs using multiple linear regression methods. The regression coefficient β was used to indicate the magnitude of causal effect. The bivariate GCA was performed on the time series of each RSN to test the causal influence from the former to the latter component. Finally, we used two sample *t*-tests to compare the ECs between patients and HCs. For multiple comparison correction, we used Bafferoni corrects as previously mentioned.

## Results

### Baseline characteristics of anti-NMDAR encephalitis patients and HCs

The detail demographic and clinical data of the anti-NMDAR encephalitis patients and HCs in this study were shown in Table 1. Statistical analysis revealed no significant differences in age, gender, Pittsburgh sleep quality index (PSQI), and education level between patients and HCs (all *p* > 0.05). Although there is no statistically significance, the scores for HDRA and HARS were higher in patients compared to HCs (*p* > 0.05). However, the MoCA score for patients was significantly lower than that of the HCs (*p* < 0.05). Patients also exhibited significant differences in WCST scores, with fewer correct numbers and correct categories, more perseverative errors and random errors compared to HCs (*p* < 0.05).

### ICA results of resting-state networks

**Figure 1:**
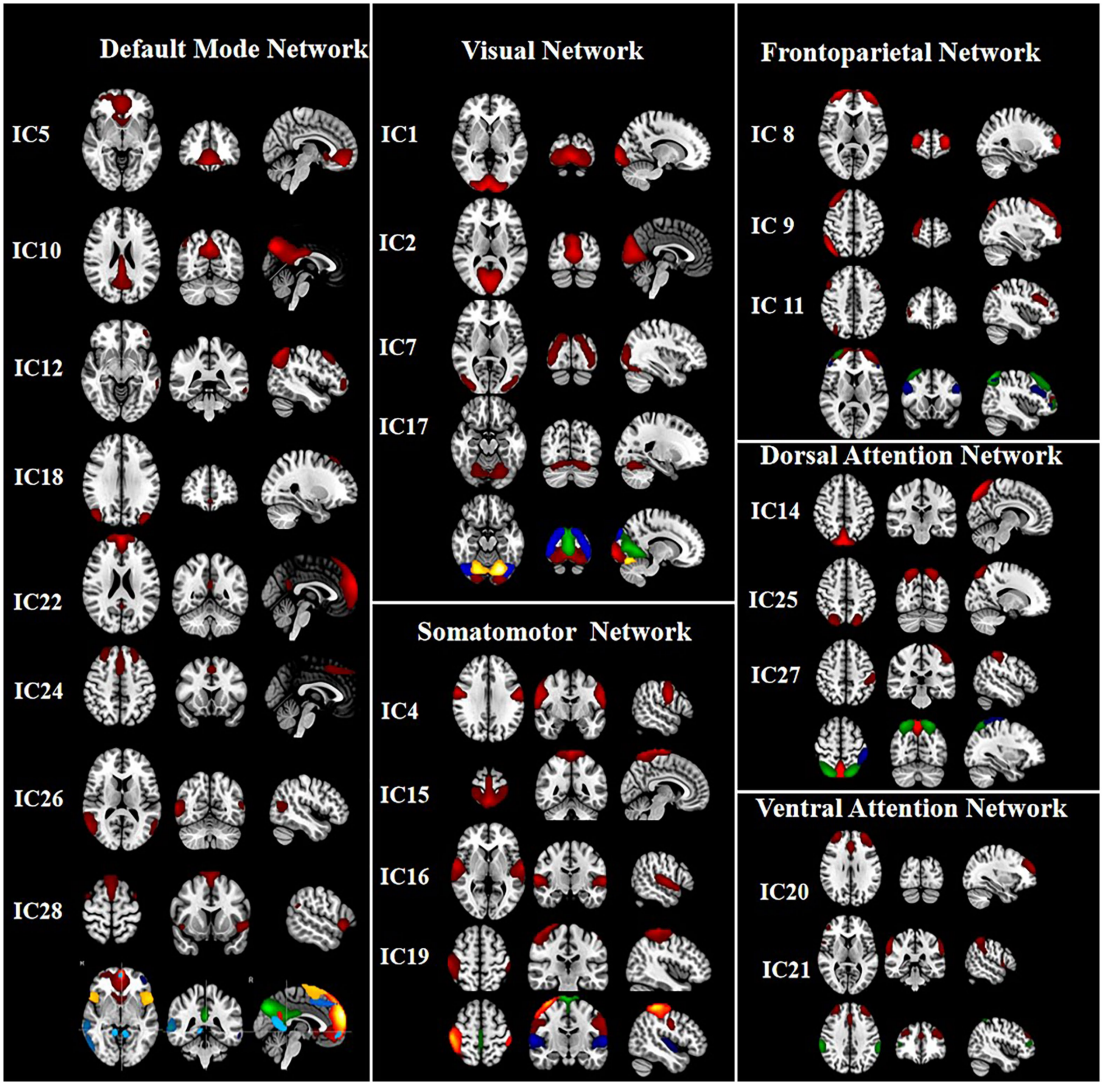
The group independent component analysis (ICA) was used to extract 31 components. Finally, 24 meaningful components were defined. The mean spatial maps of ICs across all subjects were further obtained, and the threshold was set at *p* < 0.001 to get the final spatial maps of RSNs. According to the different functional and anatomical characteristics of ICs, IC5, IC10, IC12, IC18, IC22, IC24, IC26, IC28 and IC1, IC2, IC7, IC17, and IC4, IC15, IC16, IC19, and IC8, IC9, IC11, and IC14, IC25, IC27, and IC20, IC21 were categorized into the default mode network (DMN), visual network (VN), somatomotor network (SMN), frontoparietal network (FPN), dorsal attention network (DAN), ventral attention network (VAN), respectively.

Thirty-one ICs were extracted by ICA. Twenty-four ICs (IC 1, IC 2, IC 4, IC 5, IC 7, IC 8, IC 9, IC 10, IC 11, IC 12, IC 14, IC 15, IC 16, IC 17, IC 18, IC 19, IC 20, IC 21, IC 22, IC 24, IC 25, IC 26, IC 27, and IC 28) were retained as meaningful RSNs in this study (Figure 1). According to the different functional and anatomical characteristics of ICs, they are divided into six networks, respectively. These networks are: default mode network (DMN: IC 5, IC 10, IC 12, IC 18, IC 22, IC 24, IC 26, and IC 28), visual network (VN: IC 1, IC 2, IC 7, and IC 17), somatomotor network (SMN: IC 4, IC 15, IC 16, and IC 19), dorsal attention network (DAN: IC 14, IC 25, IC 27), fronto-parietal network (FPN: IC 8, IC 9, and IC 11), ventral attention network (VAN: IC 20 and IC 21).

### Analyses of group differences in intra-FC between anti-NMDAR encephalitis and HCs within each IC

**Figure 2:**
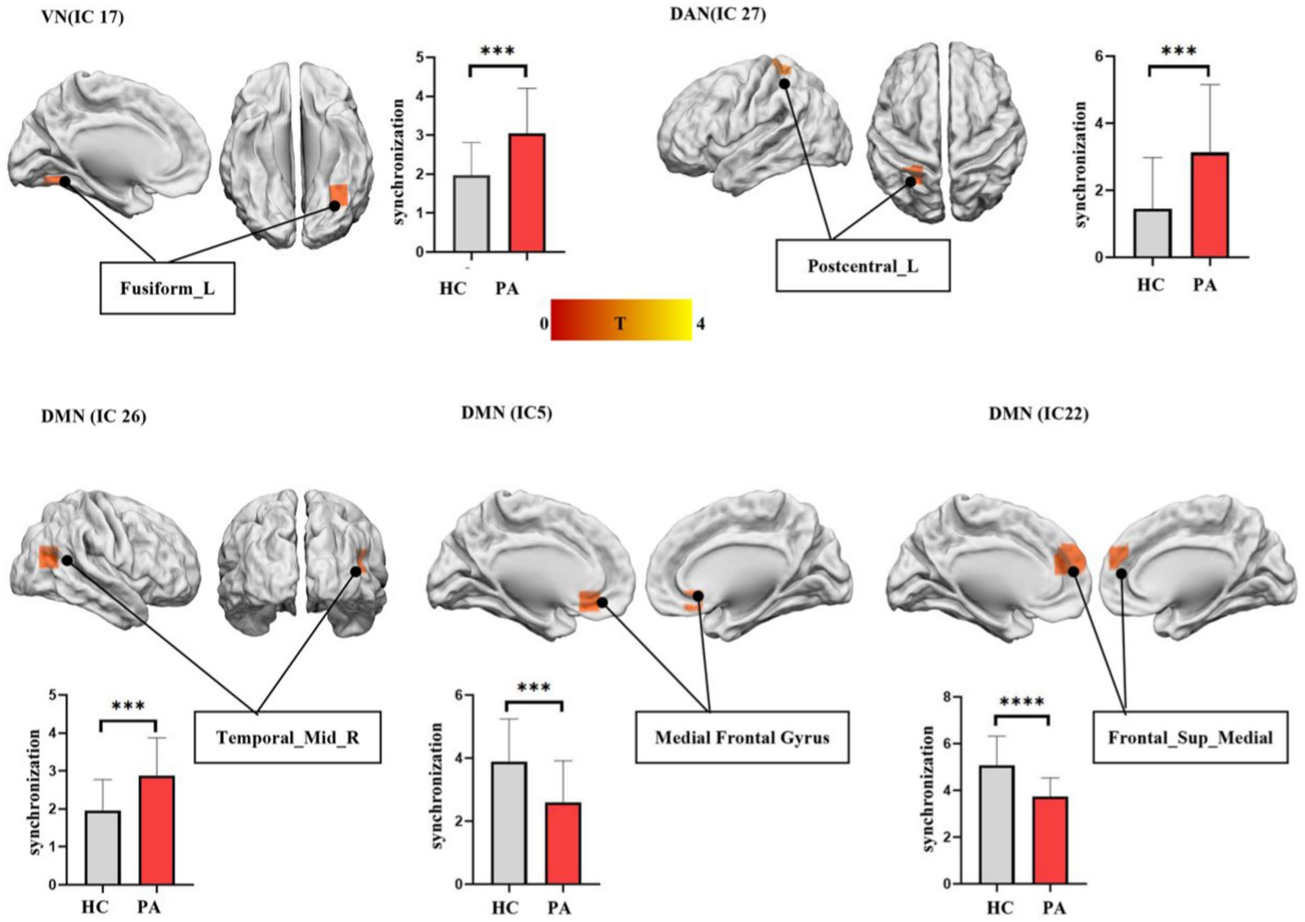
The differences in functional synchronization between anti-NMDAR encephalitis patients and healthy controls (HCs). Two-sample *t*-tests were used to identify the significant group differences in functional synchronization between anti-NMDAR encephalitis patients and HCs. The significantly decreased functional synchronization was found in the core subsystem of DMN (IC 5 and IC 22) in anti-NMDAR patients. A significantly increased functional synchronization was found in dorsal medial subsystem of DMN (IC 26), DAN (IC 27), and VN (IC 17) in anti-NMDAR patients. AlphaSim estimate was used to showing differences between the two groups within each IC, threshold at *p* < 0.05.

The significant differences in intra-FC between anti-NMDAR encephalitis patients and HCs within each IC were showed in Figure 2. Compared to HCs, the patients showed significantly decreased intra-FC within the core subsystem of DMN (IC 5 and IC 22) and increased intra-FC within dorsal medial subsystem of DMN (IC 26), DAN (IC 27) and VN (IC 17).

### Analyses of FCs

**Figure 3:**
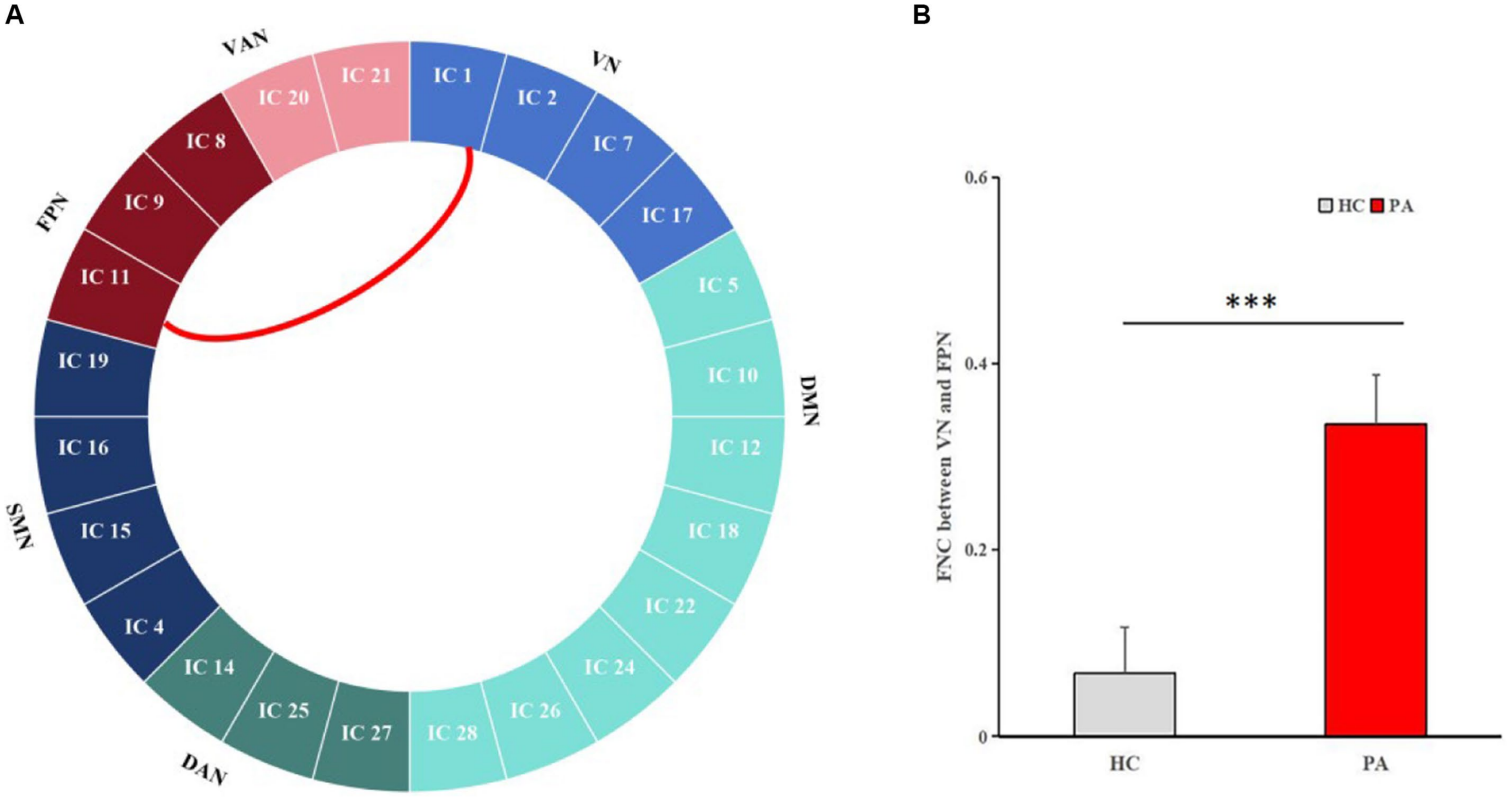
Pairwise comparisons of inter-network connectivity aberration. Comparison of inter-FC between the 24 ICs in HCs and anti-NMDAR encephalitis patients. Compared with HCs, the most significantly increased FC between VN (IC 1) and FPN (IC 11) was obtained (*p* < 0.0001, with Bonferroni correct).

The changes of the large-scale inter-FCs were identified by Pearson correlation coefficients between each pair of the 24 ICs. Compared to HCs, patients showed significantly increased inter-FCs between VN (IC 1) and FPN (IC 11) (*p* < 0.0001, with Bonferroni correct, Figure 3).

### Analyses of effective connectivities internetwork

**Figure 4:**
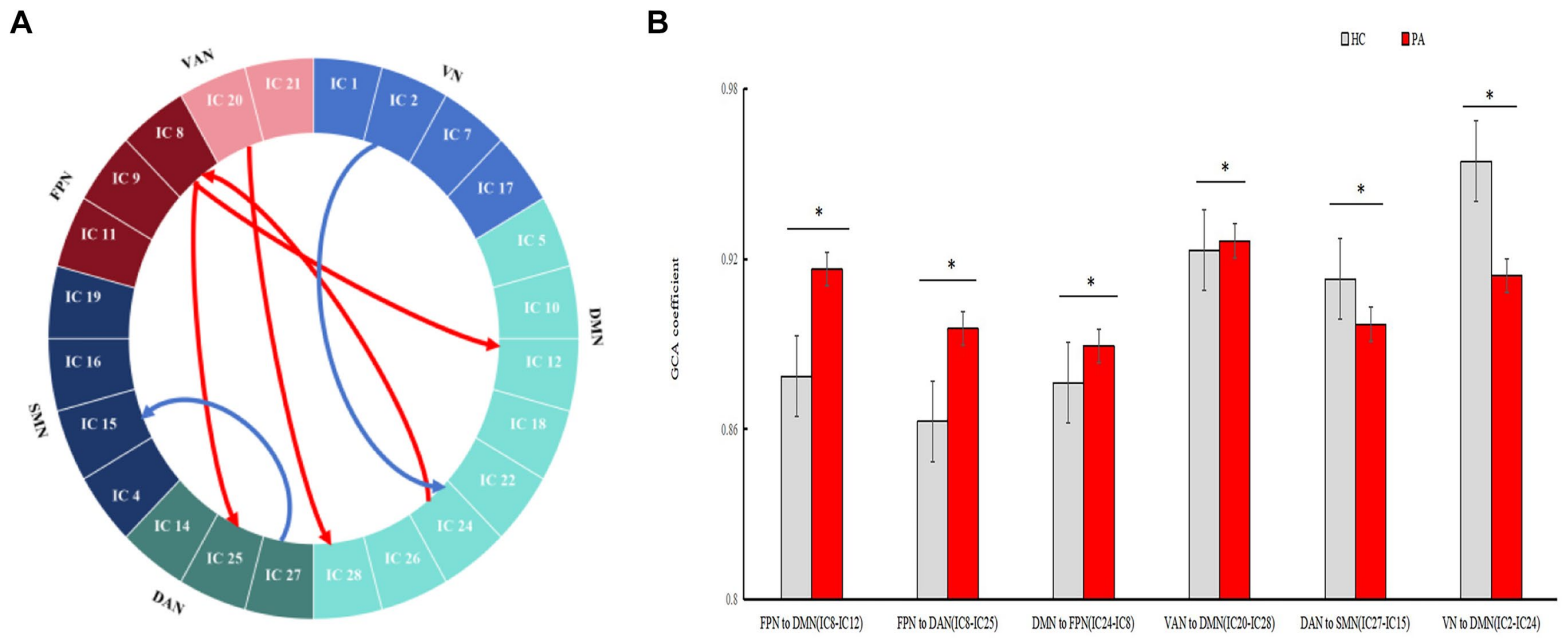
The differences in EC between ICs. Granger causality analysis (GCA) was used to calculate the effective connectivity between the 24 ICs in HCs and anti-NMDAR encephalitis patients. GCA identified significantly enhanced ECs from FPN to DMN (IC8 to IC 12), from FPN to DAN (IC8 to IC25), from DMN to FPN (IC24 to IC8), and from VAN to DMN (IC20 to IC28) in patients. Compared to HCs, the significantly decreased ECs from DAN to SMN (IC 27 to IC15), from VN to DMN (IC2 to IC 24) was showed.

In our study, compared to HCs, the enhanced ECs from FPN to DAN (IC 8 to IC 25, *p* = 0.0063), from DMN to FPN (IC 24 to IC 8, *p* = 0.0023), from FPN to DMN (IC 8 to IC 12, *p* = 0.0125), from VAN to DMN (IC 20 to IC 28, *p* = 0.0424) were found in anti-NMDAR patients. Additionally, There is significant decreased EC from VN to DMN (IC 2 to IC 24, *p* = 0.049), from DAN to SMN (IC 27 to IC 15, *p* = 0.0391) (see Figure 4).

## Discussion

The aim of our study was to investigate the intrinsic specific connectivity alterations within and between RSNs in patients with anti-NMDAR encephalitis. We found that, compared to HCs, anti-NMDAR encephalitis patients showed aberrant intra-FC within DMN, increased intra-FC within VN and PN, increased inter-FC between VN-FPN, and enhanced ECs from FPN to DAN, from DMN to FPN, from FPN to DMN, from VAN to DMN, decreased EC from VN to DMN, from DAN to SMN. These findings mentioned above provide new insights into the functional reorganization of anti-NMDAR encephalitis.

In our study, patients with anti-NMDAR encephalitis showed decreased intra-FC in the core subsystem of DMN, including medial prefrontal cortex (mPFC) and superior frontal gyrus. The superior frontal gyrus is related to emotion regulation, attention control, and decision-making, while the mPFC mainly involved in executive function and working memory (Miller and Cohen, 2001; Colgin, 2011). Decreased intra-FC of these two ICs in patients may result in downregulation of cognitive function and memory. Previous studies have indicated that patients showed decreased volume, cortical thickness, and white matter in the frontal cortex (Long et al., 2022; Xu et al., 2022). Additionally, patients showed down-regulated static amplitude of low-frequency fluctuations in the mPFC (Wu et al., 2023) and reduced regional homogeneity in the right superior frontal gyrus (Wu et al., 2022). Huang et al. (2022) observed significant changes in global network efficiency, local network efficiency, and path length of the right superior frontal gyrus in patients too. The mechanism of anti-NMDAR encephalitis involves the internalization of NMDAR induced by anti-NMDAR antibodies. This leads to a reversible reduction of NMDAR on the neuron surface and subsequent hypofunction. In addition to the hippocampus, the prefrontal cortex showed a higher density of NMDAR (Monaghan and Cotman, 1985), so it is more vulnerable to attack in anti-NMDAR encephalitis. Our results support the notion that the prefrontal cortex may be a major impaired region in anti-NMDAR encephalitis (von Schwanenflug et al., 2022). The spontaneous thoughts involves both the DMN and FPN (Smallwood et al., 2012). In the anti-NMDAR encephalitis we studied, enhanced EC from FPN to DMN may be associated with increased self-awareness. Increased intra-FC in temporal lode, as a core of dorsal medial subsystem (DMS) in DMN, was found in our study. Previous studies also reported that patients exhibited enhanced metabolism in temporal lode. In our study, the intra-FC changes of DMS and core subsystem were contrary. We speculate that this may be related to functional reorganization within the DMN. To maintain homeostasis, FC increased in the DMS to compensate for the decreased FC in the core subsystem. Animal experiments have also confirmed that when local brain function is disrupted, other brain regions play a compensatory role in order to maintain proper functioning (Chen et al., 2021). As with previous studies, our study further illustrate the importance of DMN in anti-NMDAR encephalitis.

In addition, patients also showed increased intra-FC within VN. VN includes the lingual gyrus, fusiform gyrus, and occipital cortex, which is associated with visual image processing and recognition. Previous studies have reported significant decreased FC within the VN (Peer et al., 2017). However, our study found that patients exhibited increased FC, specifically in the fusiform gyrus, which is consistent with our previous study (Guo et al., 2020). Increased local cerebral blood flow and hypermetabolism in this network is reported (Guo et al., 2020; Kerik-Rotenberg et al., 2020). Patients with anti-NMDAR encephalitis usually present with decreased memory function. To avoid the impact of these deteriorating conditions, compensatory mechanisms may be activated by enhancing intra-FC in VN. Previous studies showed that VN was differentially and dynamically coupled with FPN and DMN on the basis of task goals (Chadick and Gazzaley, 2011). In our study, FC between VN and FPN were increased, and EC was reduced from VN to DMN. The abnormalities may be related to the dysfunction of patients in processing different information. In previous studies, increased FC between FPN and VN was significantly correlated with mind-wandering errors and other behaviors (Zhou and Lei, 2018). The decreased EC from VN to DMN indicated that the influence of VN on DMN is weakened, which may explain the impaired memory function in patients despite increased VN functional compensation. Enhanced intra-FC within posterior central gyrus of DAN was shown too. Abnormal FC in the posterior central gyri is associated with violent tendencies (Tiihonen et al., 2008) and antisocial behavior (Aoki et al., 2014). Consisted with previous study (Cai et al., 2020), patients often showed psychiatric abnormalities and aggressive behavior, which may be related to intra-FC abnormalities of the posterior central gyrus.

The inter-FC and EC between these RSNs play crucial roles in the brain’s normal functioning. On the anatomy, the FPN is situated between the DMN and DAN, and interacts with them. The FPN plays a crucial role in executive attentional control and may also regulate memory and emotion through changes in FC with the DAN or DMN. We found increased EC from FPN to DAN and to DMN. It means there is aberrant EC in processing internal and external information in patients. However, the inter-FC of the above networks did not change significantly, suggesting that ECs may be more sensitive than inter-FCs. In addition, decreased EC from DAN to SMN and enhanced EC from VAN to DMN were also reported in our study. This suggests that anti-NMDAR encephalitis exist widely function networks impairment, which is related to the wide distribution of NMDAR in multiple brain regions.

There are several limitations in our study. First, the number of two groups was relatively small, resulting in a sample size mismatch between patients and HCs. Therefore, further studies are needed to replicate our findings in more patients. Second, drug use is another important confounding factor. The patient’s course of disease is not defined too. Third, the hippocampus is the region with the largest distribution of NMDAR. In our study, abnormalities in FC and EC were not observed in the hippocampus. We speculate that it may be related to the shorter course of the disease.

## Conclusion

In summary, this study confirms that patients with anti-NMDAR encephalitis exhibit abnormal intra-FC in the DMN, VN, and DAN. Additionally, the study reveals abnormal inter-FC and causal interactions between RSNs. These findings indicate that anti-NMDAR encephalitis is a disorder affecting the regulation of networks, specifically those involved in self-referential activities and visual processing. Although there is less abnormal inter-FC, more abnormal ECs is showed in anti-NMDAR encephalitis. Our study enhances understanding of the pathological mechanism underlying anti-NMDAR encephalitis.

## Data Availability

The datasets presented in this study can be found in online repositories. The names of the repository/repositories and accession number(s) can be found in the article/Supplementary material.

## Footnotes

^1^ http://www.restfmri.net/forum/DPARSF
