# Supplementary material for: Abnormal large-scale resting-state functional networks in anti-N-methyl-D-aspartate receptor encephalitis
Source: Front Neurosci. 2024 Aug 19;18:1455131. doi: 10.3389/fnins.2024.1455131 (PMC11366611; doi:10.3389/fnins.2024.1455131)
Supplement: Supplementary file 1 [file Table_1.docx]

**Supplementary table 1 Patient clinical and demographic information**

| **Patient** | **Sex** | **Age** | **Years of education(mean ± SD)** | **NMDAR-IgG Serum at onset** | **NMDAR-IgG CSF at onset** | **Moca scores** | **HARS** | **HDRS** | **PSQI** | **mRS at onset** | **Acute disease symptoms** | **Duration of acute symptoms** | **Neuro-ICU treatment** | **Tumor** | **Hormone** | **Gammaglobulin** | **Immunosuppressant** | **Other therapy** |
| --- | --- | --- | --- | --- | --- | --- | --- | --- | --- | --- | --- | --- | --- | --- | --- | --- | --- | --- |
| 1 | M | 55 | 8 | 1:10 | （-） | 21 | 10 | 5 | 7 | 0 | Psychiatric symptoms,balderdash,seizure,somnipathy | No detection | No detection | No detection | No detection | No detection | No detection | No detection |
| 2 | 2F | 35 | 8 | 1:10 | 1:10 | 23 | 1 | 1 | 0 | 0 | Seizure,hallucination,balderdash,sense of limb loss,depression | 65 | 12 | None | methylprednisolone, dexamethasone | gammaglobulin | None | Sodium valproate, levetiracetam, Clonazepam, diazepam, Lumi sodium, midazolam, ribavirin, Ganciclovir |
| 3 | M | 14 | 9 | 1:10 | （-） | 29 | 1 | 1 | 0 | 0 | Seizure,irritability,sweating,dizziness and headaches | 27 | No detection | None | No detection | No detection | No detection | No detection |
| 4 | M | 21 | 9 | 1:100 | 1:32 | 26 | 4 | 3 | 6 | 0 | Seizure,raving,overeating,irritability | 42 | 0 | None | prednisone | gammaglobulin | Rituximab | Levetiracetam, quetiapine, Olanzapine, haloperidol |
| 5 | 2F | 28 | 7 | 1:320 | 1:32 | 26 | 2 | 0 | 0 | 0 | Psychiatric symptoms,indifference and silence,emotional disturbance,somnipathy,urinary and bowel incontinence | 46 | No detection | None | No detection | No detection | No detection | No detection |
| 6 | M | 23 | 15 | 1:10 | 1:32 | 27 | 1 | 0 | 0 | 0 | Seizure,involuntary movement,indifference | 57 | 0 | None | dexamethasone, prednisone | None | None | Sodium valproate, carbamazepine, olanzapine |
| 7 | M | 18 | 11 | No detection | 1:32 | 29 | 2 | 1 | 1 | 0 | Psychiatric symptoms,gibberish,irritability,loss of immediate memory,computation,headache,vomiting | 25 | 0 | None | methylprednisolone | None | None | Ganciclovir |
| 8 | M | 16 | 10 | No detection | 1:32 | 26 | 1 | 0 | 0 | 0 | Seizure,headache,urinary and bowel incontinence,disturbance of eye movement | 44 | 10 | None | methylprednisolone | gammaglobulin | None | Sodium valproate, quetiapine, Clonazepam, Lumi sodium, Ganciclovir, ribavirin |
| 9 | F | 23 | 0 | 1:32 | 1:3.2 | 8 | 3 | 1 | 0 | 1 | Seizure,dizziness,fever,nausea and vomiting | 35 | 0 | None | dexamethasone,methylprednisolone | gammaglobulin | None | Sodium valproate, sodium lumi, clonazepam, Ganciclovir |
| 10 | M | 29 | 17 | No detection | 1:3.2 | 24 | 1 | 0 | 5 | 0 | Balderdash,irritability,seizure,irritability,irritability,diarrhea | 66 | 7 | None | methylprednisolone | gammaglobulin | None | Sodium valproate, Sodium lumina, levetiracetam, Ganciclovir, quetiapine |
| 11 | F | 43 | 4 | No detection | 1:32 | 20 | 0 | 0 | 0 | 0 | Fever,psychiatric symptoms,irritability psychiatric symptoms,disturbance of consciousness,seizure | 40 | 0 | teratoma | methylprednisolone, prednisone | gammaglobulin | None | Sodium valproate, clozapine |
| 12 | F | 29 | 14 | No detection | 1:32 | 27 | 2 | 0 | 0 | 0 | Psychiatric symptoms,seizure,balderdash,phonism,fear,somnipathy | 101 | 0 | teratoma | methylprednisolone, prednisone | gammaglobulin | None | Sodium valproate, carbamazepine, quetiapine |
| 13 | F | 31 | 8 | No detection | 1:32 | 22 | 13 | 7 | 3 | 0 | Seizure,fever and headache,balderdash,irritability | 59 | 0 | None | methylprednisolone, prednisone | gammaglobulin | cyclophosphamide | Sodium valproate, Olanzapine, quetiapine, oxazepine |
| 14 | M | 28 | 15 | No detection | 1:10 | 27 | 0 | 0 | 0 | 0 | Fever and headache,psychiatric symptoms,balderdash,irritability,disturbance of consciousness,apathy,stupor,nausea and vomiting | 76 | 0 | None | methylprednisolone, prednisone | gammaglobulin | cyclophosphamide | quetiapine |
| 15 | F | 17 | 9 | 1:100 | 1:32 | 15 | 8 | 5 | 2 | 0 | Balderdash,seizure | 33 | No detection | None | No detection | No detection | No detection | No detection |
| 16 | M | 17 | 10 | 1:100 | 1:32 | 25 | 6 | 5 | 1 | 0 | Psychiatric symptoms,hallucination,emotional disturbance,nausea and vomiting | 89 | None | None | None | gammaglobulin | azathioprine | quetiapine |
| 17 | M | 61 | 5 | （-） | 1:3.2 | 10 | 5 | 2 | 0 | 3 | Seizure | 30 | 0 | Oral squamous cell carcinoma | methylprednisolone | None | None | Sodium valproate, levetiracetam |
| 18 | M | 47 | 15 | No detection | 1:10 | 24 | 7 | 5 | 0 | 0 | Balderdash,fever,urinary incontinence,sinus tachycardia | 32 | 0 | None | methylprednisolone, prednisone | gammaglobulin | None | Levetiracetam, olanzapine |
| 19 | F | 24 | 16 | 1:100 | 1:32 | 30 | 2 | 2 | 0 | 0 | Fever,balderdash,sinus tachycardia,disturbance of consciousness | 62 | 20 | None | methylprednisolone | gammaglobulin | None | Carbamazepine |
| 20 | F | 34 | 11 | 1:100 | 1:10 | 25 | 3 | 0 | 2 | 0 | Psychiatric symptoms,seizure,apathy,irritability,involuntary facial movements | 58 | 30 | teratoma | methylprednisolone, dexamethasone | gammaglobulin | None | Sodium valproate, quetiapine, levetiracetam, carbamazepine, ribavirin, ganciclovir |
| 21 | F | 31 | 8 | 1:100 | 1:32 | 19 | 5 | 2 | 12 | 0 | Somnipathy,seizure | 49 | 0 | None | methylprednisolone | gammaglobulin | None | Sodium valproate, oxazepine, Clonazepam |
| 22 | F | 30 | 8 | 1:32 | 1:3.2 | 24 | 5 | 3 | 0 | 0 | Vomiting,limb numbness,walking instability,seizure | 32 | 0 | None | methylprednisolone, prednisone | gammaglobulin | None | Quetiapine, Olanzapine, haloperidol, ganciclovir, clonazepam |
| 23 | M | 35 | 8 | 1:32 | 1:10 | 17 | 1 | 1 | 0 | 0 | Headache,delirious,raving,uncontrollable emotions,nausea and vomiting,somnipathy,phonism | 18 | 0 | None | methylprednisolone | gammaglobulin | None | None |
| 24 | F | 44 | 5 | 1:320 | 1:100 | 24 | 2 | 0 | 0 | 0 | Seizure,decreased memory,irritability,somnipathy,delirium,gibberish,sinus retardation | 41 | 0 | None | methylprednisolone, prednisone | gammaglobulin | None | Oxcarbazepine, olanzapine |
| 25 | F | 22 | 17 | 1:32 | 1:32 | 27 | 2 | 2 | 0 | 0 | Balderdash | 24 | 0 | None | methylprednisolone，prednisone | gammaglobulin | None | Acyclovir, citicoline |

**Supplementary table 2 Wisconsin Card Sorting Test**

|  | Correct categories | Correct numbers | Random errors | Perseverative errors |
| --- | --- | --- | --- | --- |
| NM002 | 4 | 72 | 56 | 23 |
| NM003 | 3 | 66 | 62 | 35 |
| NM004 | 3 | 66 | 62 | 44 |
| NM005 | 7 | 89 | 39 | 25 |
| NM006 | 9 | 106 | 21 | 12 |
| NM007 | 7 | 102 | 26 | 15 |
| NM008 | 3 | 88 | 40 | 34 |
| NM010 | 8 | 101 | 27 | 19 |
| NM011 | 2 | 63 | 65 | 43 |
| NM012 | 9 | 100 | 15 | 10 |
| NM013 | 0 | 73 | 55 | 0 |
| NM014 | 9 | 99 | 17 | 13 |
| NM015 | 4 | 64 | 64 | 38 |
| NM016 | 4 | 79 | 49 | 29 |
| NM019 | 9 | 96 | 14 | 11 |
| NM021 | 1 | 50 | 78 | 40 |
| NM024 | 4 | 92 | 36 | 20 |
| NM027 | 9 | 101 | 13 | 11 |
|  |  |  |  |  |
| HC001 | 9 | 99 | 14 | 12 |
| HC002 | 9 | 98 | 14 | 11 |
| HC003 | 9 | 101 | 17 | 11 |
| HC004 | 9 | 97 | 16 | 11 |
| HC005 | 9 | 105 | 15 | 13 |
| HC006 | 7 | 100 | 28 | 18 |
| HC007 | 9 | 95 | 31 | 24 |
| HC008 | 7 | 99 | 29 | 11 |
| HC009 | 9 | 103 | 23 | 18 |
| HC010 | 6 | 94 | 34 | 26 |
| HC011 | 6 | 89 | 39 | 27 |
| HC012 | 6 | 99 | 29 | 15 |
| HC013 | 9 | 95 | 19 | 11 |
| HC014 | 7 | 99 | 29 | 14 |
| HC015 | 9 | 93 | 17 | 9 |
| HC016 | 8 | 109 | 19 | 13 |
| HC017 | 9 | 95 | 13 | 10 |
| HC018 | 9 | 101 | 13 | 9 |
| HC021 | 9 | 95 | 14 | 10 |
| HC024 | 9 | 99 | 16 | 10 |
| HC028 | 9 | 96 | 13 | 10 |
| HC030 | 4 | 95 | 33 | 6 |
| HC031 | 9 | 102 | 19 | 13 |

**Supplementary table 3 The ratio weight of each IC in each function network**

| **Independent component** | **VN** | **SMN** | **DAN** | **VAN** | **LN** | **FPN** | **DMN** |
| --- | --- | --- | --- | --- | --- | --- | --- |
| **IC 1** | 0.859447005 | 0 | 0 | 0 | 0 | 0 | 0 |
| **IC 2** | 0.805116279 | 0 | 0 | 0 | 0 | 0.002790698 | 0.02744186 |
| **IC 4** | 0 | 0.696588869 | 0.149910233 | 0.008976661 | 0 | 0 | 0 |
| **IC 5** | 0.105486205 | 0.04998991 | 0.075330816 | 0.065125263 | 0.041052844 | 0.074840718 | 0.153631043 |
| **IC 7** | 0.8407281 | 0 | 0.080773606 | 0 | 0 | 0 | 0 |
| **IC 8** | 0 | 0 | 0 | 0.093023256 | 0 | 0.497416021 | 0.38372093 |
| **IC 9** | 0 | 0 | 0.06006192 | 0.000619195 | 0 | 0.507120743 | 0.242724458 |
| **IC 10** | 0.12388724 | 0 | 0.017062315 | 0 | 0 | 0.305637982 | 0.395400593 |
| **IC 11** | 0 | 0 | 0.217237308 | 0 | 0 | 0.715466352 | 0.048406139 |
| **IC 12** | 0 | 0 | 0.050209205 | 0.00251046 | 0.028451883 | 0.163179916 | 0.631799163 |
| **IC 14** | 0.099526066 | 0.000947867 | 0.367772512 | 0 | 0 | 0.172511848 | 0.007582938 |
| **IC 15** | 0 | 0.446808511 | 0.221884498 | 0.005319149 | 0 | 0 | 0 |
| **IC 16** | 0 | 0.645784996 | 0 | 0.081206497 | 0 | 0 | 0.236658933 |
| **IC 17** | 0.1966759 | 0 | 0.001385042 | 0 | 0 | 0 | 0 |
| **IC 18** | 0.201886792 | 0 | 0.199056604 | 0 | 0 | 0.037735849 | 0.399056604 |
| **IC 19** | 0 | 0.428131417 | 0.305954825 | 0.001026694 | 0 | 0.016427105 | 0 |
| **IC 20** | 0 | 0 | 0 | 0.45474373 | 0 | 0.443838604 | 0.053435115 |
| **IC 21** | 0 | 0.009039548 | 0.186440678 | 0.404519774 | 0 | 0.302824859 | 0.059887006 |
| **IC 22** | 0 | 0 | 0 | 0.019503546 | 0 | 0.021276596 | 0.765957447 |
| **IC 24** | 0 | 0 | 0 | 0.343387471 | 0 | 0.102088167 | 0.515081206 |
| **IC 25** | 0.005741627 | 0 | 0.773205742 | 0 | 0 | 0.089952153 | 0 |
| **IC 26** | 0.194199243 | 0 | 0.30517024 | 0.179066835 | 0 | 0 | 0.313997478 |
| **IC 27** | 0 | 0.355140187 | 0.448598131 | 0 | 0 | 0.061682243 | 0 |
| **IC 28** | 0 | 0.010152284 | 0.042639594 | 0.291370558 | 0.001015228 | 0.012182741 | 0.318781726 |

VN: visual network, SMN: sensorimotor network, DAN: dorsal attention network, VAN: ventral attention network, LN: limbic network, FPN: frontoparietal network, DMN: default mode network,
